# Dental Use and Spending in Medicare Advantage and Traditional Medicare, 2010-2021

**DOI:** 10.1001/jamanetworkopen.2024.0401

**Published:** 2024-02-26

**Authors:** Lisa Simon, Christopher Cai

**Affiliations:** 1 Department of Medicine, Brigham and Women’s Hospital, Boston, Massachusetts

## Abstract

This cross-sectional study examines Medicare Advantage and traditional Medicare beneficiaries’ use of and spending for dental services.

## Introduction

Dental care improves health for older US adults.^1^ However, traditional Medicare (TM) rarely provides dental coverage. Up to 97% of Medicare Advantage (MA) plans offer dental coverage,^2^ but little is known about plan composition or how beneficiaries use dental services.

## Methods

This cross-sectional analysis uses 2010 to 2021 Medical Expenditure Panel Survey data analyzed between May 17 and December 30, 2023. The MassGeneral Brigham institutional review board determined this study to be non–human participant research and waived the need for informed consent. The study follows the STROBE reporting guideline.

Included respondents were enrolled in TM or MA based on end-of-year response or in both Medicaid and Medicare based on having Medicaid for any month in the year. We tabulated annual dental visits and expenditures stratified by insurance type, adjusting to 2021 dollars. Dental visits were classified as preventive, restorative, or emergency. We conducted 2-sided *t* and Pearson χ^2^ tests to assess demographic differences between MA and TM, with *P* < .05 considered significant, and regressions to account for respondent demographics identified as varying substantively between TM and MA beneficiaries (age, sex, race and ethnicity, region, income, dual eligibility status), evaluating differences in dental use and costs. We ran negative binomial regressions for counts and a regression of log-dental cost to account for skew from higher dental expenses. Analyses were performed using Stata, version 15.1 (StataCorp LLC). The eMethods in Supplement 1 provide further details.

## Results

Our sample comprised 60 695 adults (MA vs TM mean [95% CI]: age, 71.7 [71.4-71.9] vs 70.4 [70.1-70.7] years; 56.3% [55.5%-57.1%] vs 53.5% [52.9%-54.2%] female; 1.8% [1.6%-2.2%] vs 2.7% [2.2%-3.2%] Alaska Native, American Indian, or other identity; 4.4% [3.6%-5.3%] vs 3.2% [2.6%-3.8%] Asian; 10.6% [9.6%-11.6%] vs 9.8% [8.9%-10.8%] Black; 11.0% [9.9%-12.2%] vs 6.2% [5.5%-6.9%] Hispanic; 72.2% [70.6%-73.9%] vs 78.2% [76.8%-79.6%] White; income, 384.6% [374.0%-395.3%] vs 431.9% [419.7%-444.0%] of federal poverty level). In 2010 to 2021, MA patients were more likely to delay dental care due to cost (unadjusted difference, 2.8%; 95% CI, 1.4%-4.1%; *P* < .001) but not for any reason and had similar total annual dental visits and likelihood of preventive, restorative, or emergency visits as TM beneficiaries; lower out-of-pocket (OOP) costs (−$26; 95% CI, −$50 to −$2; *P* = .04), though not significant in adjusted analyses; and no differences in OOP costs per visit or total costs in adjusted or unadjusted analyses (Table). Expenditures and use varied by year (Figure), but differences between MA and TM did not change over time. Regressions accounting for differential enrollment in MA by race and ethnicity, income, and dual eligibility for all dental use and cost measures did not reveal significant differences between MA and TM.

**Table.** Dental Service Use^a^

Variable	Value (95% CI)		Unadjusted difference		Adjusted difference^b^	
	MA (n = 23 846)	TM (n = 36 849)	MA − TM (95% CI)	*P* value	MA − TM (95% CI)	*P* value
Total dental visits, No. annually	1.2 (1.2 to 1.2)	1.2 (1.2 to 1.3)	−0.1 (−0.1 to 0.00)	.08	0.00 (−0.05 to 0.05)	.92
Dental care, %^c^						
Any	46.5 (45.2 to 47.8)	47.6 (46.5 to 48.7)	−1.1 (−2.6 to 0.4)	.14	0.7 (−0.6 to 2.0)	.28
Any preventive	42.2 (40.8 to 43.5)	43.4 (42.2 to 44.5)	−1.2 (−2.7 to 0.3)	.11	0.7 (−0.7 to 2.0)	.33
Any restorative	18.7 (17.9 to 19.5)	19.0 (18.4 to 19.7)	−0.3 (−1.3 to 0.7)	.51	0.4 (−0.6 to 1.3)	.41
Any emergency	5.7 (5.4 to 6.1)	5.8 (5.5 to 6.1)	0.0 (−0.5 to 0.4)	.86	0.0 (−0.5 to 0.5)	>.99
Total expenditures, 2021 US dollars	485 (458 to 511)	502 (477 to 527)	−17 (−47 to 12)	.25	−2 (−30 to 27)	.91
Total OOP costs, 2021 US dollars	302 (282 to 323)	328 (307 to 349)	−26 (−50 to −2)	.04	−16 (−40 to 7)	.17
Total OOP costs per visit, 2021 US dollars	222 (211 to 233)	217 (207 to 227)	5 (−8 to 19)	.44	0.8 (−13 to 15)	.90
Delay in necessary care, %^d^	4.5 (4.0 to 5.1)	4.2 (3.8 to 4.6)	0.4 (−0.2 to 0.9)	.21	0.4 (−0.1 to 0.9)	.15
Delay in any care due to cost, %^e^	16.1 (15.0 to 17.2)	13.3 (12.4 to 14.3)	2.8 (1.4 to 4.1)	<.001	2.3 (1.0 to 3.6)	<.001

Abbreviations: MA, Medicare Advantage; OOP, out-of-pocket; TM, traditional Medicare.

^a^ Data are presented as weighted percentage estimates (95% CIs), unless otherwise specified.

^b^ Adjusted for age, sex, race and ethnicity, region, and income (adjustment methods provided in the eMethods in Supplement 1).

^c^ Preventive visits were defined as routine cleaning, examinations, fluoride treatment, or radiographs; restorative as crowns, root canals, implants, bridges, or fillings; and emergencies as oral surgery, abscess, or extractions.

^d^ Medicare Expenditure Panel Survey respondents in years 2010 to 2017 were asked if they delayed any necessary dental care in the past calendar year.

^e^ Medicare Expenditure Panel Survey respondents in years 2018 to 2021 were asked if they delayed any dental care due to cost in the past calendar year.

**Figure.**
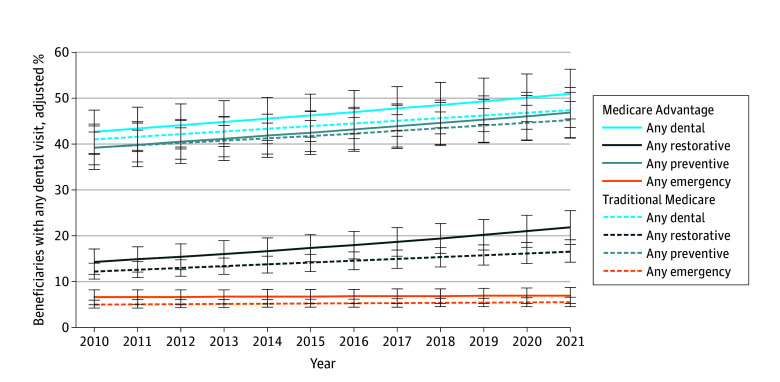
Trends in Dental Use and Spending, Medicare Advantage vs Traditional Medicare, 2010-2021 Data are presented as weighted percentage estimates (95% CIs) accounting for income, sex, region, percentage of beneficiaries with Medicaid, and race and ethnicity. Adjustment methods are provided in the eMethods in Supplement 1.

## Discussion

We found that MA and TM beneficiaries had similarly low rates of dental use. Lack of access to needed dental care may explain why adverse dental outcomes increase as individuals transition from private insurance to Medicare.^3^

The majority of Medicare beneficiaries are now enrolled in MA, which costs 17% more per capita than TM due to favorable selection and intensive diagnostic coding.^4^ The MA plans justify this cost by offering benefits such as dental, but use management, limited networks, and high cost sharing may restrict patients’ actual use of these benefits.

Our findings suggest that MA beneficiaries delay dental care due to cost at higher rates and do not have rates of preventive dental use, suggesting that low use may not be exclusively associated with restricting low-value services but with, eg, restrictive networks, high OOP costs, or lack of awareness of benefits.^5^ The Centers for Medicare & Medicaid Services propose mandating that MA insurers send beneficiaries a mid-year letter describing their unused benefits, which could increase dental use.^6^ Dental use and expenditure are limited by self-report and potential recall bias. Medicare policy must focus on enhancing dental access for both MA and TM beneficiaries; the additional cost of MA does not appear to be offset by superior dental use.

## Notes

Supplement 1.
eMethods.

eReferences

Supplement 2.
Data Sharing Statement
