# Error in Table

**DOI:** 10.1001/jamanetworkopen.2024.36713

**Published:** 2024-09-03

In the Research Letter titled “Dental Use and Spending in Medicare Advantage and Traditional Medicare, 2010-2021,”^1^ published February 26, 2024, the *P* value for the adjusted difference for “any emergency” was incorrectly reported as <.001 in the Table. The correct *P* value is >.99. This article has been corrected.^1^
