# Supplementary material for: Dental Use and Spending in Medicare Advantage and Traditional Medicare, 2010-2021
Source: JAMA Netw Open. 2024 Feb 26;7(2):e240401. doi: 10.1001/jamanetworkopen.2024.0401 (PMC10897735; doi:10.1001/jamanetworkopen.2024.0401)
Supplement: Supplement 1. — eMethods. eReferences [file jamanetwopen-e240401-s001.pdf]

Supplemental Online Content

Simon L, Cai C. Dental use and spending in Medicare Advantage and traditional Medicare, 2010-2021. *JAMA Netw Open*. 2024;7(2):e240401. doi:10.1001/jamanetworkopen.2024.0401

eMethods
eReferences

This supplemental material has been provided by the authors to give readers additional information about their work.

© 2024 Simon L et al. *JAMA Network Open.*

eMethods

Background on the MEPS

The Medical Expenditure Panel Survey (MEPS) is a nationally representative survey of healthcare utilization among non-institutionalized US adults. MEPS includes a subset of respondents to the National Health Interview Survey who are interviewed five times over the course of 30 months (Panels in 2020 and 2021 were extended to nine rounds due to the COVID-19 pandemic). Our annual estimates thus represent responses from two to three panels being collected over the course of the calendar year. Racial/ethnic identity in MEPS is obtained via self-identification. Individuals identified as "other" in our data include those who identified as American Indian or Alaska Native, or as a racial/ethnic identity other than White, Black, Hispanic (written as Latinx in our manuscript), or Asian American. Gender is also collected by self-report.

Due to survey design, only respondents who indicate a private insurance plan are asked if their plan covers several benefits, including dental insurance. Thus public insurance recipients, including all patients on Medicare, are not asked if they have dental insurance. For this reason we describe oral health outcomes and dental service use, but do not include dental insurance as a measure.

Limitations of our analysis include that survey demographics were self-reported and subject to recall bias. Additionally, we conducted cross-sectional analyses and were not able to assess longitudinal trends.

Additional data on dental service use was obtained from the 2010-2021 Dental Visit Files, which includes both expenditure and type of dental procedure received. Expenditure data in MEPS is verified by discussion and review of receipts with interviewees as well as adjudication with the Medical Provider Component that includes information from health systems. All surveys are available on the MEPS website maintained by the Agency for Healthcare Research and Quality. [1] Responses must be weighted to account for complex survey weights.

Software and IRB Approval

Analyses were conducted using Stata, version 15.1 (StataCorp), accounting for complex survey sampling and MEPS-provided weights to generate nationally representative estimates. Our reporting meets the Strengthening the Reporting of Observational Studies in Epidemiology (STROBE) guidelines. Our study was determined to be not human subjects research by the MassGeneral Brigham Human Subjects Review Board (IRB23-0445).

Study Variables

In the MEPS 2010-2021 files, 396,024 total individuals are sampled. Of those, 8,260 (2.1%) had missing values for insurance and were excluded. Of those remaining, 60,695 reported having Medicaid and Medicare, Traditional Medicare without Medicaid or Medicare Advantage without Medicaid and were included in the primary analysis. We included all respondents who indicated they were enrolled in Medicare at the end of the year of study. We were unable to evaluate the role of supplemental insurance or Medigap plans because MEPS does not include information about supplemental private insurance plans and, as noted above, Medicare beneficiaries are not asked whether they have dental insurance. We tabulated annual dental visits and expenditures stratified by insurance type (MA vs TM). Costs include charges billed to insurance as well as out-of-pocket expenses paid by the respondent that are verified through evaluation of receipts, respondent report, and insurance reporting. We used the Consumer Price Index (CPI) for out-of-pocket visits and the Gross Domestic Product (GDP) for total

© 2024 Simon L et al. *JAMA Network Open.*

expenditures to adjust to 2021 dollars as per guidelines recommended by the MEPS.[2] We classified visits as preventative (routine cleaning, examinations, fluoride or radiographs), restorative (crowns, root canals, implants, bridges, or fillings) or emergencies (oral surgery, abscess, or extractions) based on clinical gestalt and our prior work.[3]

We defined Traditional Medicare versus Medicare Advantage using a variable corresponding to insurance enrollment at the end of the calendar year. To better reflect the high churn among Medicaid and Medicare (Dual Eligible) beneficiaries we define Dual Eligible as having any Medicaid in addition to year end Medicare in our primary analysis.

Analyses

For descriptive statistics, we computed two-sided t-tests for continuous variables. For categorical variables, we report p values from Pearson's X^2 testing. For our outcome variables, we first report unadjusted means with t-tests for difference in mean between MA and TM. We repeated these regressions adjusted for age, gender, racial and ethnic identity, region, and income (defined as percent of the federal poverty level). For binary outcomes (e.g. percentage of respondents having a dental visit), we conducted negative binomial regressions to account for zero inflation. For dental expenditure outcomes, we ran a regression with the log of cost as the outcome to account for potential skew from individuals with higher dental expenses. As sensitivity analyses, we ran regressions that included interaction variables between year and enrollment in Medicare Advantage, as well as regressions with interaction variables between racial and ethnic identity and Medicare Advantage enrollment and dual eligibility and Medicare Advantage enrollment. These were not significant.

To calculate adjusted means, we used negative binomial regression for utilization data, as utilization of healthcare is zero inflated and right skewed. For expenditure data, which is also highly zero inflated and right skewed, we used a two-part model. We used a probit model to estimate the probability any individual had any healthcare expenditures and a generalized linear model for the subset of individuals with expenditures. We used a log link and gamma distribution as has been done in prior studies with expenditure data with the MEPS.[4] We then calculated adjusted means for different outcome variables (any dental visit, any preventive dental visit, any restorative dental visit and any emergency visit) or the magnitude of expenditures, holding independent variables (income, sex, region, percent of beneficiaries with Medicaid, race/ethnicity) at the their means. For categorical variables, we created dummy variables for each outcome (e.g. for the categorical variable "sex," we created a variable for each response in the MEPS: probability of male sex, probably of female sex.) and held these at their means in our adjusted means of our outcome measures.

eReferences

1. Agency for Healthcare Research and Quality. Medical Expenditure Panel Survey. Published online August 26, 2009. Accessed December 21, 2023. https://meps.ahrq.gov/mepsweb/survey_comp/survey_questionnaires.jsp
2. *USING APPROPRIATE PRICE INDICES FOR ANALYSES OF HEALTH CARE EXPENDITURES OR INCOME ACROSS MULTIPLE YEARS*. Agency for Healthcare Research and Quality; 2023. Accessed December 21, 2023. https://meps.ahrq.gov/about_meps/Price_Index.shtml
3. Simon L, Song Z, Barnett ML. Dental Services Use: Medicare Beneficiaries Experience Immediate And Long-Term Reductions After Enrollment: Study examines dental services use

© 2024 Simon L et al. *JAMA Network Open.*

by Medicare beneficiaries. *Health Aff (Millwood)*. 2023;42(2):286-295. doi:10.1377/hlthaff.2021.01899
4. Deb P, Norton EC. Modeling Health Care Expenditures and Use. *Annu Rev Public Health*. 2018;39(1):489-505. doi:10.1146/annurev-publhealth-040617-013517

© 2024 Simon L et al. *JAMA Network Open.*
