# Supplementary material for: Dental Use and Spending in Medicare Advantage and Traditional Medicare, 2010-2021
Source: JAMA Netw Open. 2024 Feb 26;7(2):e240401. doi: 10.1001/jamanetworkopen.2024.0401 (PMC10897735; doi:10.1001/jamanetworkopen.2024.0401)
Supplement: Supplement 2. — Data Sharing Statement [file jamanetwopen-e240401-s002.pdf]

Data Sharing Statement

Simon. Dental Use and Spending in Medicare Advantage and Traditional Medicare, 2010-2021. *JAMA Netw Open*. Published February 26, 2024.
doi:10.1001/jamanetworkopen.2024.0401

Data
Data available: Yes
Data types: Data (not involving human participants)
How to access data: All data is available publicly on the MEPS website:
https://meps.ahrq.gov/mepsweb/data_stats/download_data_files.jsp
When available: With publication

Supporting Documents
Document types: Statistical/analytic code
How to access documents: Please email corresponding author Lisa Simon MD DMD at lsimon@bwh.harvard.edu for all Stata code.
When available: With publication

Additional Information
Who can access the data: Anyone requesting the data can access it publicly.
Types of analyses: All data is publicly available for analysis.
Mechanisms of data availability: Data is available without investigator support needed. Statistical code will be made available without investigator support.
